# A Dual-Bandpass Frequency Selective Absorber with Wide-Angle Oblique Incidence

**DOI:** 10.3390/ma18030473

**Published:** 2025-01-21

**Authors:** Yong-Xing Che, Qiang Sun, Xue-Mei Du, Yong-Ling Ban

**Affiliations:** 1 School of Electronic Science and Engineering, University of Electronic Science and Technology of China, Chengdu 611730, China; byl@uestc.edu.cn; 2 National Key Laboratory of Scattering and Radiation, Beijing 100854, China; m18810187525@163.com; 3 School of Information Science and Technology, Southwest Jiaotong University, Chengdu 610031, China; sunqiang@swjtu.edu.cn

**Keywords:** absorber, dual-bandpass, CA, wide-angle oblique incidence

## Abstract

This study proposes a frequency-selective absorber (FSA) with dual passbands and wide-angle oblique incidence. The design consists of a circuit analog (CA) sheet and a dual-bandpass frequency selective surface (FSS) sheet, both embedded in dielectric slabs separated by a foam spacer. The CA sheet unit cell is based on a tripole loaded with multiple shorted transmission lines and lumped resistors. In this way, the performance of the CA sheet is equivalent to a resistive sheet in a low-frequency band and a transparent sheet in two high-frequency bands. By comprehensively designing the CA sheet and the dual-bandpass FSS sheet, we created an FSA structure that exhibits microwave absorption in the band from 2.6 GHz to 9.2 GHz with a reflectivity lower than −10 dB. It also possesses transmission in the 12.2–15.1 GHz and 30.6–31.5 GHz bands, with a transmittance greater than −3 dB in both. In addition, the FSA structure provides a stable transmission response of up to 60° of oblique incidence and absorption performance of up to 45° of oblique incidence in TE and TM polarization. A 400 × 400 mm flat FSA sample was fabricated, was measured, and is discussed. The experimental results are consistent with the simulation results, proving that the proposed FSA design holds great potential for applications in dual-frequency low-scattering radomes with high curvature and multi-directional electromagnetic interference suppression.

## 1. Introduction

Frequency-selective absorbers (FSAs) with passbands are multi-sheet integrated periodical structures with absorption and transmission qualities, usually comprising a CA sheet and a bandpass FSS sheet [1,2]. FSAs can be used as radomes, absorbing electromagnetic waves at certain frequency bands to reduce detectability while remaining transparent at the frequency of radars, thus not affecting antenna signal transmission. FSAs can also mitigate electromagnetic interference between shared-aperture antennas or interference from external electromagnetic signals. The FSA, compared to the common frequency-selective surface (FSS), possesses absorptive capabilities in the out-of-band. As demand for radomes with low radar cross-sections and electromagnetic interference suppression has grown, this technology has gained increasing attention from researchers [3,4].

Several techniques have been proposed in the literature for an optimal FSA with passband designs. In [3], the equivalent circuit approximation method for multi-sheet absorbing structures and an analysis method based on transmission line theory were provided. An integrated absorbing and transmitting structure design based on a resistive film design was proposed, achieving low-frequency transmission and high-frequency absorption. In [5], an FSS loaded with lumped resistors was proposed, achieving low-frequency broadband absorption and high-frequency bandpass performance. In [6,7], novel periodic electromagnetic structures were proposed in which waves are transmitted in the middle-frequency band and absorbed on both sides of the passband. However, the structure is a single polarization structure, and the transition effect between the absorption band and the passband needs to be strengthened. Based on metal square rings, a polarization-insensitive structure was designed in [8], achieving polarization stability in oblique incidence angles and reducing passband insertion loss. However, the maximum incidence angle is 30°. In [9], a method using loading lumped resistors and LC resonators to design an FSA structure was presented, which had very low sensitivity with respect to the polarization and incidence angles of EM waves. In [10], a reconfigurable periodic electromagnetic structure with passband switching characteristics was proposed. The design uses PIN diodes loaded in the bottom sheet of the bandpass periodic electromagnetic structure, and the passband can switch between transmission and total reflection by switching the PIN diode. Similarly, the transparent part of the structure is polarization-sensitive.

With the emergence of multi-band signal transmission requirements such as dual-/multi-band common aperture antennas, FSRs with multiple transmission bands are needed to integrate transmission and absorption. A few multi-band FSR designs with loaded lumped resistors have been proposed in the literature [11,12,13,14,15,16,17,18,19]. The literature presents various structures for the different positional relationships of absorbing bands (A) and transmitting bands (T) in the frequency domain, such as A-T-A-T [11,12,14,16,19] and A-T-A-T-A [13,15,17,18]. These studies primarily focus on design methods for the normal incidence of electromagnetic waves and adopt symmetric structural designs to achieve polarization insensitivity. In [11,12,14,15,16,17,18,19], performance under oblique incidence was calculated and tested, but no targeted design for angular insensitivity was provided. The maximum incidence angle at which the performance remains stable was 40°. In [11,12,13], it was shown that large unit spacing in the structures can easily lead to grating lobes at large incidence angles, making it impossible to achieve stable transmission. In [15], to miniaturize the unit and eliminate the CA sheet unit’s influence on the higher passband, capacitive components were added, resulting in a very narrow transmission bandwidth. In [16], a cascaded metasurface design was adopted to achieve absorption between two transmission bands, but the transmission bandwidth was significantly affected and narrow. In [17], more metasurface layers were designed to expand the transmission bandwidth, but the structural thickness increased significantly, reaching 0.15 × λ_L_ (where λ_L_ corresponds to the lowest absorption frequency).

In the aforementioned literature on FSAs, the small incidence angle of electromagnetic waves poses significant challenges for curved radomes and electromagnetic shielding that require transmission and absorption over a wide-angle range. To address these limitations, this study introduces an FSA based on inductive loads, exhibiting low insertion loss within a dual-bandpass and supporting a maximum incidence angle of up to 60°.

A double-sheet, angle-stable, and polarization-insensitive FSA for dual-band applications is proposed to overcome the issue of dual-bandpass stability. An equivalent unit cell circuit model is mathematically analyzed for normal incidence. The design procedure and measurement results for the fabricated FSA sample are presented and discussed as follows, proving that the structure has resonance stability with respect to different polarization and incident angles.

## 2. Design of Dual-Bandpass FSA

Dielectric sheets and CA sheets have specific roles in the performance of an absorber. By adjusting the thickness and permittivity of the dielectric sheets and optimizing the geometry parameters and resistance of the CA unit cell, good absorption can be achieved. To design an FSA that can absorb at a certain frequency range while remaining transparent in the two higher-frequency ranges, the ground plane must be transparent in those ranges. This can be achieved by making the ground plane a dual-bandpass FSS. Meanwhile, the CA sheet must be “invisible” at these transparent frequencies, but they should otherwise be able to absorb.

A novel tripole based on a traditional tripole element loaded with lumped resistors was designed as the CA sheet unit cell, arrayed in a triangular grid. There are two advantages to using a tripole when designing a CA sheet unit cell [1]: the first is that it can meet the dual polarization operation, and the second is that it can be arranged more compactly, delaying the onset frequency or incident angle of the grating lobes and meeting the requirements for a large incident angle and high-frequency-band applications. To meet the requirements of low-frequency resonance, the size of a tripole unit cell must be relatively large when applying it to an FSA structure with low-frequency absorption and high-frequency transmission, thus generating second harmonic resonance at high frequencies. If the second harmonic resonance is excited in the high-frequency transmission band, it will deteriorate the transmission performance of the structure.

To eliminate the influence of the second harmonic resonance of the CA sheet on high-frequency transmissions, three shorted twin transmission lines (STTLs) and two shorted four-wire transmission lines (SFTLs) are attached to each leg of the traditional tripole as an inductive load to shorten the tripole, separating the fundamental and second harmonic resonances. When implemented, the shorted transmission line allows the length of the dipole to be shortened. It does not affect the resonant frequency for the second harmonic resonance. For the fundamental resonance, the shorted transmission line functions as an inductor. For the second harmonic resonance, the current loops use the transmission line as a capacitive end load to make the effective length of the leg of the tripole half of the wavelength. This can achieve a higher second-order resonance frequency while keeping the low-frequency resonance band constant, leaving enough space for the two transmission passbands.

**Figure 1:**
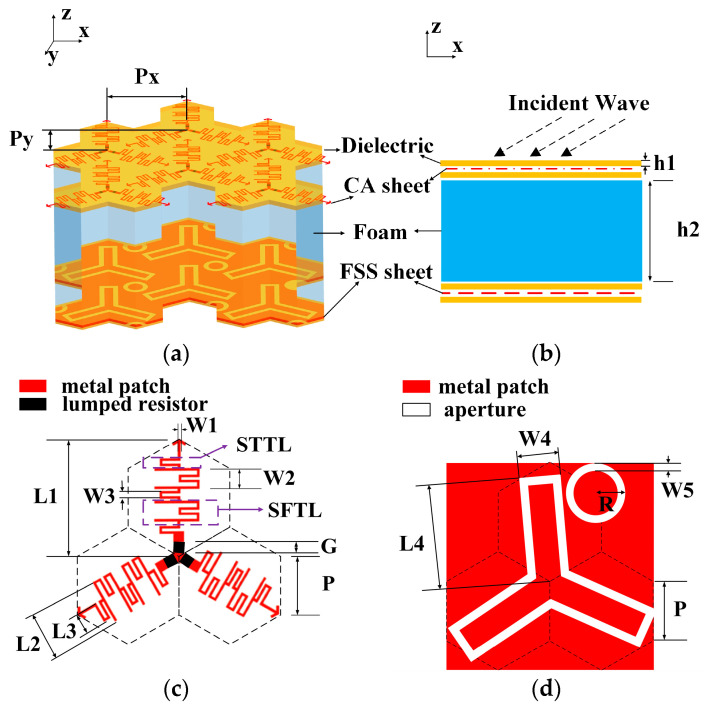
Geometry of proposed FSA: (**a**) isometric view; (**b**) side view; (**c**) unit cell of CA sheet (top sheet); (**d**) unit cell of FSS sheet (bottom sheet).

Figure 1a,b show the geometry and arrangement of the FSA, comprising dielectric slabs (ε_r_ = 4.2; tan δ = 0.01), a CA sheet, and a backed dual-bandpass FSS sheet with a foam spacer (ε_r_ = 1.1; tan δ = 0.005) in between them. Both sides of the CA and FSS sheets are covered by dielectric plates. The thickness of the dielectric slab and foam are h1 = 0.2 mm and h2 = 9 mm, respectively. The periods of the structure in the x- and y-directions are Px = 1.5 × P and Py = Px × $\sqrt{3}$. A top view of the CA sheet unit cell with optimized parameters is illustrated in Figure 1c. Resistors are loaded at the intersect point of each leg, and to achieve a wider absorption band, the optimized values of the resistors are 150 Ω. Figure 1b shows a top view of the FSS sheet unit cell, comprising two units—a tripole loop slot and a circular slot—arranged and combined on the same plane. By optimizing their structural parameters separately, a dual-bandpass is achieved. Placing circular slot units between the tripole loop slot units in the array can achieve a tight arrangement, reduce the array period, and improve performance stability at wide incident angles. The physical and geometrical parameters of this design are presented in Table 1.

**Figure 2:**
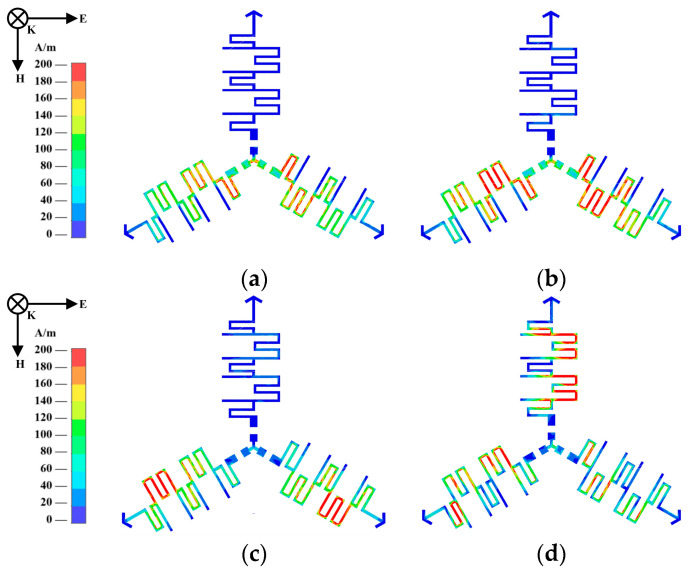
Simulated surface current of proposed unit cell at (**a**) 3.7 GHz; (**b**) 8 GHz; (**c**) 13.2 GHz; and (**d**) 31 GHz.

To further describe the working mechanism of the shorted transmission lines, the surface current distribution is shown in Figure 2. The surface current distribution was simulated with the commercial High-Frequency Structure Simulator software. ANSYS HFSS v19.0 All current distributions shown in Figure 2 were excited on the CA layer metal unit when TM-polarized electromagnetic waves were normally incident. Two typical frequencies in the absorption band and two typical frequencies that correspond to the lower and upper passbands were chosen for observation at 3.7, 8, 13.2, and 31 GHz, respectively. The transmission bands appeared at 13.2 and 31 GHz, whereas the two high-absorption frequencies could be observed at 3.7 and 8 GHz. Notably, at 13.2 GHz, the current was mainly distributed in the SFTL, whereas at 31 GHz, the current was merely concentrated in the STTL and SFTL, while the current on the metal strips connecting the lumped resistors was weak. The lower passband was determined by the SFTL, while the higher passband was jointly determined by the SFTL and STTL. Figure 2a,b show that the induct current was distributed through two branches on the leg of the tripole that were nearly parallel to the direction of the electric field, and the current in the metal strips connecting the lumped resistors was strong. Therefore, each meander line on the legs of the tripole was in resonance, playing a crucial role in achieving absorption in lower frequency bands. Moreover, lumped resistors could efficiently dissipate induced currents and achieve strong incident electromagnetic wave absorption.

**Figure 3:**
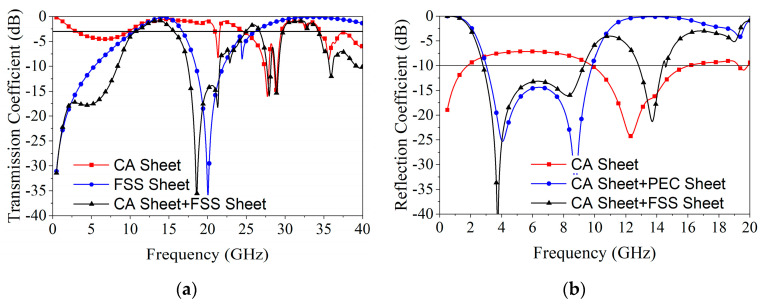
Simulated transmission and reflection coefficient under individual sheets and different combinations: (**a**) transmission coefficient; (**b**) reflection coefficient.

As shown in Figure 3, frequency response curves obtained through an HFSS simulation of individual sheets and combinations of these sheets under the normal incidence of electromagnetic waves verify the above work. The CA sheet exhibited absorption characteristics in bands from 2.6 to 9.2 GHz and transmission characteristics at 10.5 to 20 GHz and 29.8 to 34 GHz. The FSS sheet exhibited reflection characteristics in the entire frequency band below 9.2 GHz and dual-bandpass characteristics in the higher bands. The composite structure—comprising a CA sheet and a PEC sheet stacked at a quarter wavelength distance apart—enhanced the absorption performance due to resonance. By replacing the PEC sheet with an FSS sheet, both absorption and transparency were maintained. However, Figure 3b shows that near the passband, due to the insufficient reflection performance of the FSS layer, there was a slight loss in absorption.

The transmission coefficient curve of the combined structure of the CA sheet and FSS sheet shows that the FSS layer had a high transmittance, and the combined structure completely maintained the bandpass characteristics of the CA sheet, especially in the higher passband. Therefore, excellent CA layer dual-bandpass performance is the key to achieving dual-bandpass performance in FSA structures.

**Figure 4:**
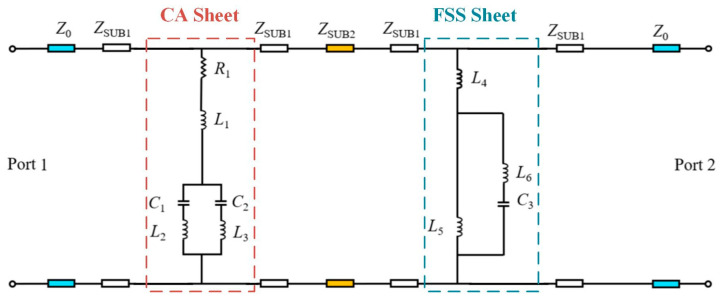
Equivalent circuit of the FSR (L_1_ = 1.07 nH; R_1_ = 255 ohm; C_1_ = 0.1421 pF; L_2_ = 3.6062 nH; C_2_ = 0.022 pF; L_3_ = 0.01 nH; L_4_ = 0.2 nH; C_3_ = 0.075 pF; L_5_ = 0.953 nH; and L_6_ = 0.1 nH).

The FSA’s equivalent circuit is illustrated in Figure 4. In the equivalent circuit, the series R_1_-L_1_ refers to the leg of the tripole with lumped resistors on the upper surface of the CA sheet. The STTL and SFTL are equivalent to C_1_-L_2_ and C_2_-L_3_, blocking the resonant response at about 14 GHz and 31 GHz, respectively. The tripole loop slot and the circular slot array of the FSS sheet are modeled as the series connection of L_6_ and C_3_ paralleled to L_5_. The metal connection between the unit cells of the FSS sheet is represented by L_4_. The equivalent impedance of the dielectric layer is represented by Z_SUB1_, and the equivalent impedance of the foam layer is represented by Z_SUB2_.

The impedance values of the CA sheet and FSS sheet, respectively, are

(1) $$Z_{CA}=R_{1}+j\omega L_{1}+\left[\left(j\omega L_{2}+1/j\omega C_{1}\right)||\left(j\omega L_{3}+1/j\omega C_{2}\right)\right]\;Z_{FSS}=j\omega L_{4}+\left[\left(j\omega L_{5}\right)||\left(j\omega L_{6}+1/j\omega C_{3}\right)\right]$$

Then, the ABCD matrices for each layer are

(2) $$A_{1}=A_{SUB1}\left[\begin{array}{cc} 1 & 0\\ \frac{1}{Z_{CA}} & 1 \end{array}\right]A_{SUB1}\;A_{2}=\left[\begin{array}{cc} {\cos\;\theta }_{\mathrm{SUB}2} & jZ_{\mathrm{SUB}2}\sin\theta_{\mathrm{SUB}2}\\ j\frac{{\sin\;\theta }_{\mathrm{SUB}2}}{Z_{\mathrm{SUB}2}} & {\cos\;\theta }_{\mathrm{SUB}2} \end{array}\right]\;A_{3}=A_{SUB1}\left[\begin{array}{cc} 1 & 0\\ \frac{1}{Z_{FSS}} & 1 \end{array}\right]A_{SUB1}$$

where

(3) $$A_{SUB1}=\left[\begin{array}{cc} {\cos\;\theta }_{\mathrm{SUB}1} & jZ_{\mathrm{SUB}1}\sin\theta_{\mathrm{SUB}1}\\ j\frac{{\sin\;\theta }_{\mathrm{SUB}1}}{Z_{\mathrm{SUB}1}} & {\cos\;\theta }_{\mathrm{SUB}1} \end{array}\right]$$

(4) $$\begin{array}{cc} \theta_{\mathrm{SUB}i}=\beta_{i}h_{i}, & i=1,2. \end{array}$$

Therefore, the total cascade matrix of the element is

(5) $$\left[\begin{array}{cc} A & B\\ C & D \end{array}\right]=A_{1}A_{2}A_{3}$$

We can normalize the above equation:

(6) $$\left[\begin{array}{cc} a & b\\ c & d \end{array}\right]=\left[\begin{array}{cc} A & \frac{B}{Z_{0}}\\ CZ_{0} & D \end{array}\right]$$

Subsequently, the reflection and transmission coefficient of the equivalent circuit model can be derived as follows:

(7) $$S_{11}=\frac{a+b-c-d}{a+b+c+d}$$

(8) $$S_{21}=\frac{2}{a+b+c+d}$$

**Figure 5:**
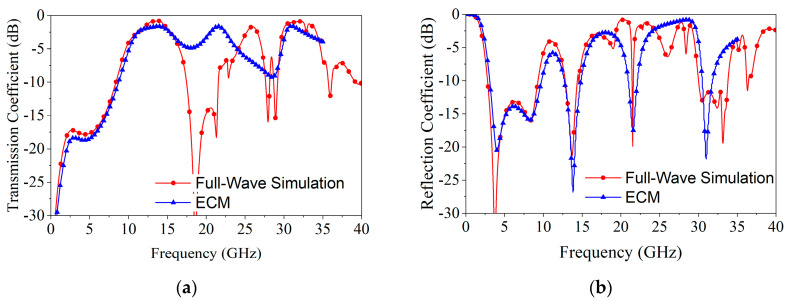
Transmission and reflection coefficients obtained through HFSS simulation and ECM calculation for normal incidence: (**a**) transmission coefficient; (**b**) reflection coefficient.

Figure 5 depicts the corresponding reflection and transmission coefficients, with good agreement between the ECM and full-wave simulation results in the absorption band and dual-passband.

**Figure 6:**
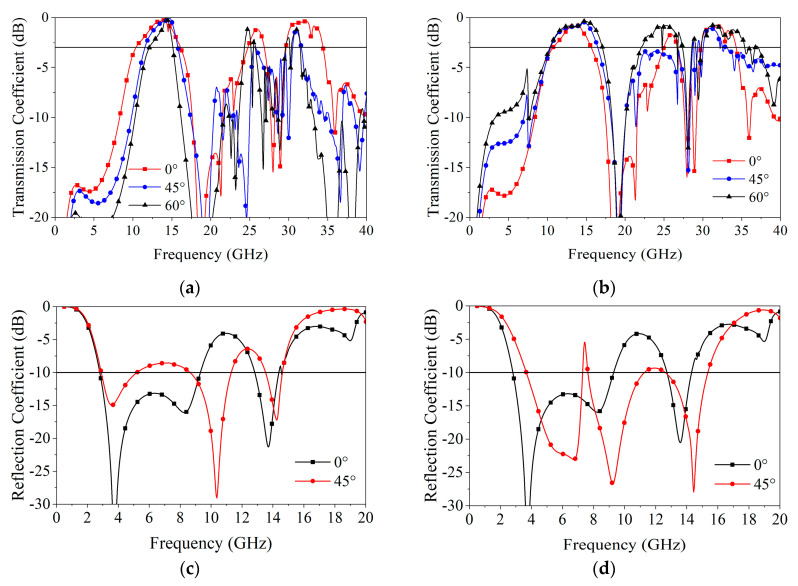
Simulated transmission and reflection coefficient under different incident angles: (**a**,**c**) TE polarization; (**b**,**d**) TM polarization.

The frequency response curves of the reflection and transmission coefficients at different incident angles of TE and TM polarization are shown in Figure 6a–d. The frequency response of the transmission band maintains good stability at large angles, except for the narrowing of the higher passband in TE polarization. This is because the response frequency band of the SFTL narrows at large angles, while the CA sheet unit generates resonance at high frequencies at large angles, which damages the higher passband.

As the incident angle of electromagnetic waves varies, unwanted unit resonance and grating lobes within the passband become the two main factors affecting wave transmission. As the incident angle increases, the frequency at which grating lobes occur shifts toward lower frequencies, damaging the passband’s transmission performance. According to a formula in [2], to avoid generating grating lobes in a triangular array arrangement at a frequency of 31 GHz and an incident angle of 60°, the spacing between the centers of the unit cells should be approximately d = 7.95 mm or less. The distance can be calculated as follows:

(9) $$d=2/3(1.15\times \lambda_{h})/(1+sin\;\theta )$$

where $\lambda_{h}=c/f_{h}$; θ=60°; c is the speed of light in vacuum; and $f_{h}\;$ is the vacuum operating frequency.

Due to the STTL and SFTL, the CA unit arrangement becomes more compact. As shown in Figure 1c, the spacing between the centers of adjacent units in the FSA structure is 3 × P = 7.5 mm, which satisfies the condition for not generating grating lobes. Therefore, even at an incident angle of 60°, as shown in Figure 6a,b, it still exhibits excellent wave transmission performance. Additionally, the unit cells are designed with one-third rotational symmetry, resulting in better polarization insensitivity.

The STTL and SFTL suppress the loss of surface-induced current due to lumped resistance in the CA sheet near 13.2 GHz and 31 GHz, enabling the incident electromagnetic waves in the frequency bands around these two frequencies to efficiently pass through the CA sheet, thus ensuring the high transmittance of the FSA structure.

Notably, the absorption performance deteriorates significantly at large angles due to a mismatch between the CA sheet unit and free space as the angle increases.

## 3. Measurement and Discussion

**Figure 7:**
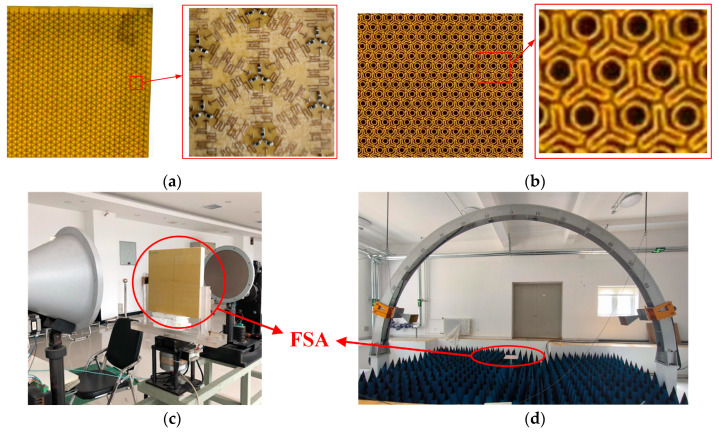
Photographs of the fabricated FSA: (**a**) details of the CA sheet; (**b**) details of the FSS sheet; (**c**) the setup for the transmission performance measurement; (**d**) the setup for the reflection performance measurement.

Using the design results in Section 2, a 400 × 400 mm FSR sample was fabricated. The metal patterns of the FSS sheet and the CA sheet were constructed on a polyimide-coated copper film using standard photochemical etching techniques, and thick film chip resistors (UR, 0402) with a resistance tolerance of ±1% were mounted using manual welding techniques. The copper layer of the polyimide-coated copper film had a thickness of 0.018 mm, and the polyimide layer (ε_r_ = 4.2; tan δ = 0.005) had a thickness of 0.02 mm. The dielectric slabs were made of epoxy resin glass fiber cloth (ε_r_ = 4.2; tan δ = 0.01), thermally pressed for curing. The foam material used for isolation between the FSS sheet and the CA sheet was PMI with low dielectric loss (ε_r_ = 1.1; tan δ = 0.005). The reflection testing of the fabricated sample was performed with the arch method. The absorber planar was horizontally placed on a foam support square plate. Changes in the incident angle could be obtained by rotating the transmitting and receiving horn antennas simultaneously. Figure 7a,b present the measured results for the oblique incidence angles. The properties were acceptable under TE and TM polarization waves until the incident angle increased to 45°. The transmission testing of the sample was performed using lens antennas. The FSA was illuminated by the narrow Gaussian beam of the lens, reducing the edge diffraction effect.

**Figure 8:**
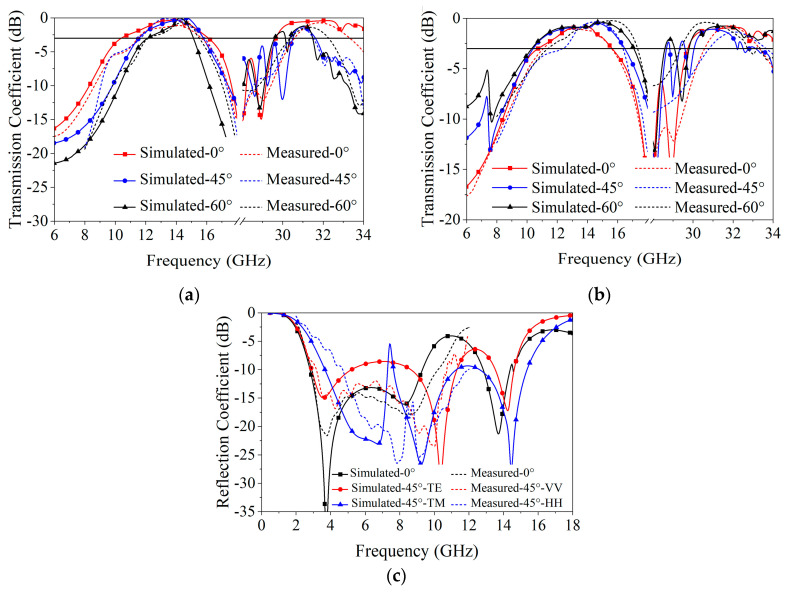
Comparison of simulated and measured S-parameters for various incidence angles: (**a**) transmission coefficient—TE polarization; (**b**) transmission coefficient—TM polarization; (**c**) reflection coefficient.

The measured reflectivity and transmittance for the FSA under various incidence angles are shown in Figure 8; for comparison, the results of the full-wave simulation from Figure 6 are also shown. The reflectivity was less than −10 dB at 2.6 GHz to 9.2 GHz, and the transmittance was greater than −3 dB at 12.2–15.1 and 30.6–31.5 GHz, with minimal insertion losses (ILs) of 0.13 dB at 13.17 GHz and 0.87 dB at 31.5 GHz. Although there was a small absorption reduction in the resonant frequencies due to the tolerance introduced during fabrication and the uncertainty in the resistance value of the CA sheet, there is good agreement between simulated and measured results.

To demonstrate the performance of our proposed FSR, comparisons with other reports are listed in Table 2. Our FSR has better angular stability, thinner thickness, and the widest relative absorption bandwidth. Compared to the designs in [12,13,14,15,16,17,18,19], our FSR has fewer lumped elements. The structure possesses more significant comprehensive performance advantages and is more suitable for practical applications, such as curved surface conformal integration and interference suppression in complex electromagnetic environments.

## 4. Conclusions

This study presented a dual-bandpass FSA with a transmission band in the Ku/Ka-band and an absorption band in the S/C/X-band. An ECM model was developed to analyze the resonance behavior of the absorber. The design offered stable transmission for incidence angles of up to 60° and absorption for incidence angles of up to 45° for TE and TM polarization. The transmission bandwidth was shown to reach 2.9 GHz in the Ku-band and 0.9 GHz in the Ka-band. The simulated results were in good agreement with the measurement results. The significant improvements of the proposed FSA include dual-passband, high absorption, low insertion loss, and a compact structure.

Compared to other FSAs [11,12,13,14,15,16,17,18,19], the proposed FSA has fewer lumped resistors, a thinner thickness, angle insensitivity, ultra-wideband absorption, and easy fabrication, making it promising for applications in radomes and anti-electromagnetic interference.

This FSA design is applicable to application scenarios with common conical or cylindrical external structures, providing dual-band signal transmission and out-of-band rejection over a wider angle range. Subsequent efforts will focus on analyzing the impact of element curvature on the performance of the FSA for curved surfaces, optimizing the FSA’s design, enhancing its absorbing performance at large incidence angles, and improving its applicability to curved surfaces.

## Figures and Tables

**Table 1:** Physical and geometrical parameters of CA sheet.

Parameter	P	L1	L2	L3	L4
Value	2.5 mm	5.5 mm	2.2 mm	1 mm	2.8 mm
Parameter	W1	W2	W3	W4	W5
Value	0.1 mm	1 mm	0.36 mm	1.7 mm	0.5 mm
Parameter	W2	W3	G	R	
Value	1 mm	0.36 mm	0.25 mm	150 Ω	

**Table 2:** Performance comparison of dual-bandpass FSAs.

Ref.	Lower Transmission Band/IL	Higher Transmission Band/IL	Absorption Band	Thickness (λ_L_)	Lumped Elements in One Unit	Angular Stability
[11]	22.2% (5.6–7 GHz)/ 0.5 dB at 6.2 GHz	18.3% (9.3–11.2 GHz)/ 0.4 dB at 10.3 GHz	40.4% (3.3–5 GHz) 19.6% (7.4–9 GHz)	0.097	3	30 (simulation)
[12]	8.9% (7.7–8.4 GHz)/ 0.39 dB at 11.9 GHz	10.7% (11.4–12.7 GHz)/ 0.64 dB at 8 GHz	47.5% (4.5–7.3 GHz) 25.6% (8.5–11 GHz)	0.116	4	30
[13]	11.1% (8.5–9.5 GHz)/ 0.43 dB at 9.1 GHz	12.4% (10.6–12 GHz)/ 0.35 dB at 11.2 GHz	45.1% (3.7–5.9 GHz) 5.0% (9.8–10.3 GHz) 16.3% (13.2–15.5 GHz)	0.068	4	0
[14]	12.3% (3.3–3.7 GHz)/ 0.24 dB at 3.5 GHz	5.5% (4.8–5 GHz)/ 0.1 dB at 4.9 GHz	56.3% (1.7–3 GHz) 20% (3.9–4.7 GHz)	0.076	4	40
[15]	7.8% (3.4–3.7 GHz)/ 1.2 dB at 3.6 GHz	7.8% (4.9–5.3 GHz)/ 0.83 dB at 5.05 GHz	32.9% (2–2.8 GHz) 15.3% (3.8–4.4 GHz) 29.9% (6.3–8.5 GHz)	0.077	12	40
[16]	8% (10.8–11.7 GHz)/ 0.56 dB at 11.5 GHz	8.2% (16.3–17.7 GHz)/ 0.52 dB at 17.5 GHz	89.4% (3.9–10.2 GHz) 14.3% (13–15 GHz)	0.082	4	30
[17]	46% (6.9–11 GHz)/ 0.44 dB at 8 GHz	23.3% (13.7–17.3 GHz)/ 0.41 dB at 15.4 GHz	81.1% (2–4.8 GHz) 13.7% (11.5–13.2 GHz) 3.4% (18.7–19.3 GHz)	0.15	8	30
[18]	7% (7.8–8.4 GHz)/ 0.76 dB at 8.1 GHz	11.8% (9.6–10.8 GHz)/ 0.52 dB at 10.2 GHz	83% (3.1–7.4 GHz) 9% (8.6–9.3 GHz) 58% (12.1–20.5 GHz)	0.08	8	30
[19]	8.6% (11.2–12.2 GHz)/ 1.0 dB at 11.7 GHz	8.8% (15.3–16.7 GHz)/ 0.7 dB at 16 GHz	95.7% (3.7–10.5 GHz) 14.7% (12.6–14.6 GHz)	0.08	4	40
This work	21.2% (12.2–15.1 GHz)/ 0.13 dB at 13.17 GHz	2.9% (30.6–31.5 GHz)/ 0.87 dB at 31.5 GHz	111.9% (2.6–9.2 GHz)	0.085	3	60 (Transmission)/ 45 (Absorption)

## Data Availability

The original contributions presented in this study are included in the article. Further inquiries can be directed to the corresponding author.
